# Is There a Natural, Non-addictive, and Non-anti-reward, Safe, Gene-based Solution to Treat Reward Deficiency Syndrome? KB220 Variants vs GLP-1 Analogs

**Published:** 2024-05-20

**Authors:** Edward Justin Modestino, Abdalla Bowirrat, David Baron, Panayotis K. Thanos, Colin Hanna, Debasis Bagchi, Eric R. Braverman, Catherine A. Dennen, Rajendra D. Badgaiyan, Aryeh R. Pollack, Kai-Uwe Lewandrowski, Alireza Sharafshah, Mark S. Gold, Kenneth Blum

**Affiliations:** 1 Department of Psychology, Curry College, Milton, USA; 2 Department of Molecular Biology, Adelson School of Medicine, Ariel University, Israel; 3 Division of Addiction Research and Education, Center for Sports, Exercise, and Mental Health, Western University of Health Sciences, Pomona, USA; 4 Department of Pharmacology and Toxicology, Jacobs School of Medicine and Biosciences, State University of New York Buffalo, USA; 5 Department of Pharmaceutical Sciences, Texas Southern University College of Pharmacy and Health Sciences, Houston, USA; 6 Division of Nutrigenomics, The Kenneth Blum Neurogenetic and Behavioral Institute, LLC, Austin, USA; 7 Department of Family Medicine, Jefferson Health Northeast, Philadelphia, USA; 8 Division of Psychiatry, Mt. Sinai School of Medicine, New York, USA; 9 Department of Psychiatry, Case University School of Medicine, Cleveland, USA; 10 Division of Personalized Pain Medicine and Education, Center for Advanced Spine Care of Southern Arizona, Tucson, USA; 11 Department of Orthopedics, Fundación Universitario Sanitas, Bogotá, D.C., Colombia; 12 Department of Orthopedics, Universidad Federal do Estado do Rio de Janeiro, Brazil; 13 Division of Genetics, Department of Cell and Molecular Biology and Microbiology, School of Science and Biotechnology, University of Isfahan, Isfahan, Iran; 14 Department of Psychiatry, Washington University, St. Louis, USA; 15 Department of Psychiatry, Vermont University School of Medicine, Burlington, USA

**Keywords:** Reward deficiency syndrome, KB220, GLP-1, Dopamine, Addiction

## Abstract

Reward deficiency syndrome (RDS) is an umbrella term encompassing a wide array of addictive behaviors that affect individuals across diverse spectra of society. Our research group has conducted a plethora of studies investigating the utilization of KB220 and its various iterations for addressing RDS, including: dopamine homeostasis, brain areas associated with dopamine, functional connectivity, qEEG, reductions of cravings, relapse prevention and detoxification, opioid-seeking and attenuation of intake, binge-drinking and withdrawal, driving under the influence (DUI), shopping and hoarding behaviors, memory decline, nightmares, paraphilias, attention deficit hyperactivity disorder (ADHD), eating disorders and weight loss, anger and stress reduction, and genetically customized compounds. In this review, we compare studies using KB220 (and variants) for these things with GLP-1 analogs. We suggest that KB220 (and its variants) demonstrate superiority over GLP-1 analogs for addressing all these issues, as evidenced by various reasons outlined herein, particularly their impact on the brain’s reward cascade and dopamine homeostasis, all while avoiding antagonism of the reward system.

## Introduction

The Neurogeneticist Kenneth Blum, Ph.D., DHL, with Dr. Ernest Noble of UCLA, published the first study to associate an allele of the D2-dopamine receptor gene (DRD2) and severe alcoholism in JAMA in 1990 [1]. This research confirmed an association preempted by a theoretical construct that identified the neurotransmitter interaction leading to dopamine release in the mesolimbic pathway as the “Brain Reward Cascade” in 1989 [2]. Earlier basic research had identified the role endorphins played in alcoholism and the blocking effect of naloxone, an opioid antagonist, in alcohol dependence, which supported the concept of a common mechanism for opiates and alcohol [3]. Blum et al. also put forward the concept of genetically induced RDS, which is a “hypodopaminergic” trait and/or state, and developed nutraceutical therapy (KB220) to treat RDS. Subsequently, Blum et al. developed the genetic addiction risk severity (GARS^®^) test to identify one’s genetic risk for RDS [4, 5] (Table 1).

**Figure 1:**
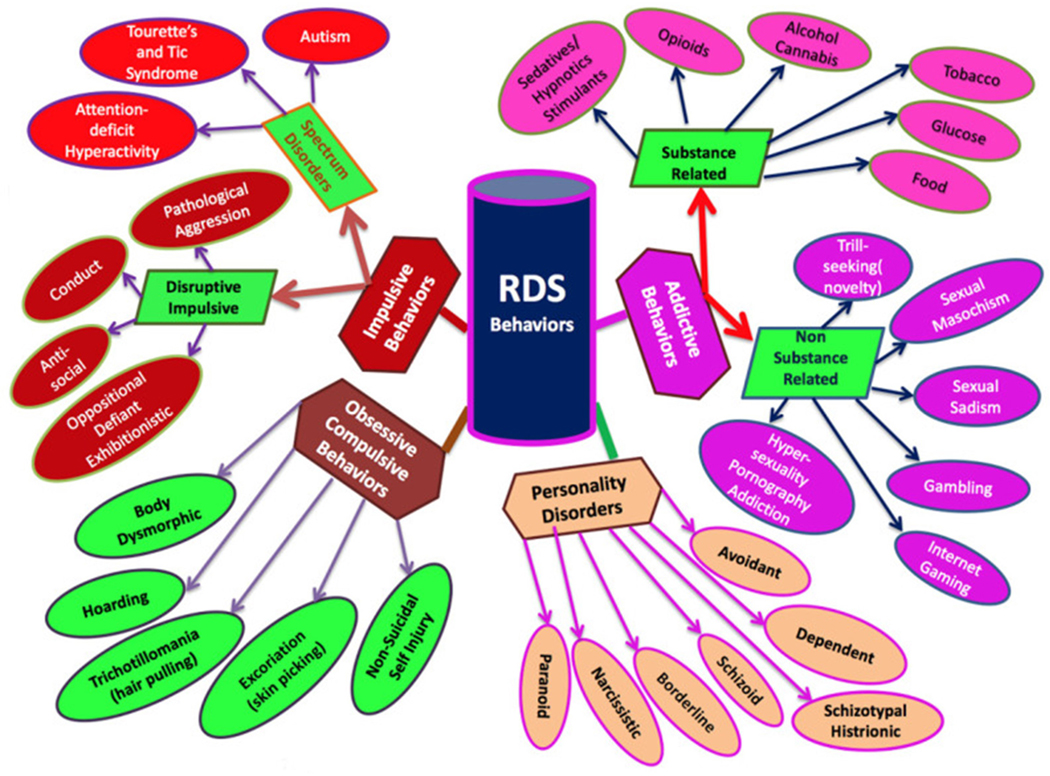
A schematic of RDS.

In the experience of Blum et al. even casual conversations of anecdotes from clinicians with knowledge of RDS with their colleagues, friends, family, and even acquaintances have divulged the ubiquitousness of subtypes of RDS (Figure 1) [7].

This figure illustrates the categorization of RDS behaviors, showing which behaviors are addictive, impulsive, obsessive, and personality disorders. Subcategories divide addictive behaviors into substance and non-substance-related and impulsive into disruptive/impulsive and spectrum disorders. These behaviors have dopaminergic dysfunction in common: acute, excess, or chronic deficit of dopamine release in the brain reward circuitry. Figure 1 and the corresponding caption has been reprinted here with permission from Blum et al. [7].

Within our research group’s studies, relapsed rates in the group using the compound KB220 (and variants) have been significantly lower with regard to the use of cocaine, opioids, alcohol, and even food-related addictive behaviors (Table 2).

Approximately 90% of those with alcohol use disorder experience at least a single relapse within the first four years following initial treatment. The relapse rates for opioid use disorders and tobacco use disorders are similar, which alludes that addictive behaviors share cognitive, behavioral, and biochemical components. As with alcohol use disorders, AUD various opioid use disorders show a greater relapse rate, greater than 80%, in patients receiving only behavioral treatments.

Our research group, as well as other researchers, have reported relapse rates based on statistics from NIDA/NIAAA. This is in accordance with the data across the scientific/medical literature, which has divulged a 76% rate.

The evaluation of relapse data is presented in table 2. was calculated from our database from studies that took place between the years 2000 and 2005. Inclusion in the presented data required that participants received a minimum of a three-month provision of the oral form of KB220.

RDS is the dysfunction of the brain reward pathway [14]. The syndrome appears to be due to dysfunction of the dopamine: acutely with an excess, chronically with a deficit in release [15]. RDS encompasses both drug and non-drug addictive behaviors and impulsive and compulsive behaviors [16]. Notably, at least 35 animal and human research studies are now published in PubMed-referenced journals (Table 3).

Currently, there is excitement related to the agonistic stimulation of GLP1 peptide and many studies showing novel benefits in RDS behaviors, including obesity, diabetes, and even substance use disorders (SUD) [17]. While we believe genetic addiction risk testing could have heuristic value in the future, the primary mechanism of action of stimulating GLP1 results in augmentation of Gamma-aminobutyric acid (GABA) transmission as well as an increase in the DAT1 function with tantamount increase in synaptic dopamine reabsorption [18, 19]. This concern is especially in those individuals who carry DNA antecedents that set up an individual at birth to have a hypodopaminergic risk [20] (Table 3).

## Discussion

Globally, in 2020, about 35 million people suffer from SUDs [73] and 280 million humans suffer from AUD [74]. Most compelling is the fact that the World Health Organization (WHO) projected that the global harmful qualities of alcohol use and abuse account for approximately 5% of premature fatalities. As such, WHO notes that alcohol use and abuse are leading causes of preventable fatalities worldwide. These nefarious outcomes are linked to consumers of alcohol having a lower rate of treatment than other psychiatric disorders [75]. The AUD-related high premature mortality rate is also linked to dopamine dysregulation, including the DRD2 A1 allele [76]. Specifically, the Taq1A polymorphism of the dopamine D2 receptor (DRD2) gene has been comprehensively studied in relation to alcoholism, is over-represented in alcohol-de-pendent individuals, and is associated with a highly increased mortality rate in alcohol-dependent individuals [77].

Along similar lines of reasoning, the United Nations Publication [73] has estimated that 19 million humans use cocaine, and about 5 million of these people that meet the criteria for cocaine use disorder (CUD), with in many cases ending in death by overdoses. This statistic is even morphed by the unfortunate fact that 58 million humans used opioids in 2018 [74], a phenomenon that is accompanied by a very high societal cost [78]. Additionally, coupled with alcohol, although tobacco use is one of the highest preventable causes of premature deaths 10.6 humans still ended up dying due to tobacco-related diseases annually [79].

While in 1995 Blum’s group coined the term RDS, in terms of reward deficiency there have been a plethora of globally based articles independently since 1995 [80–132] both positive and negative. In addition, there is a large body of articles encouraging the induction of “dopamine homeostasis” to overcome the entire reward dysregulation dilemma with its pre-addiction DNA polymorphic antecedents [133–197]. Moreover, there is strong evidence for the role of dopaminergic function or its modulation concerning mental health across one’s lifespan [198–247]. There is also a large body of published evidence revealing a remarkable sharing of common genetic po; polymorphisms with psychiatric disorders [248–268]. Finally, the role of endogenous opioids and enkephalinase inhibition in terms of reward behavior and disruptive addictive-like seeking has been formerly revealed in many published articles [269–296].

## Conclusion

Understanding these essential events and subsequent evidence, we are herein proposing that a natural, non-addicting, and non-anti-reward safe solution to treat RDS would be highly welcomed as another tool to assist in the development of inducing “dopamine homeostatic therapy” (DHT) using KB220.

## Figures and Tables

**Table 1:** Reward deficiency syndrome behaviors.

ADDICTIVE BEHAVIORS		IMPULSIVE BEHAVIORS		OBSESSIVE COMPULSIVE BEHAVIORS	PERSONALITY DISORDERS
Substance Related	Non-Substance Related	Spectrum Disorders	Disruptive Impulsive		
Alcohol	Thrill-seeking (novelty)	Attention-deficit Hyperactivity	Anti-social	Body Dysmorphic	Paranoid
Cannabis	Sexual Sadism	Tourette and Tic Syndrome	Conduct	Hoarding	Schizoid
Opioids	Sexual Masochism	Autism	Intermittent Explosive	Trichotillomania (hair pulling)	Borderline
Sedatives/Hypnotics	Hypersexual		Oppositional Defiant	Excoriation (skin picking)	Schizotypal
Stimulants	Gambling		Exhibitionistic	Non-suicidal Self-Injury	Histrionic
Tobacco	Internet Gaming				Narcissistic
Glucose					Avoidant
Food					Dependent

* Table 1 has been reprinted here with permission from Blum et al. [6,7].

**Table 2:** KB220 (and variants compounds) Relapse Rates versus Control Participants in those with Reward Deficiency Syndrome (RDS).

Patient Category	KB220 Relapse Rate (N) (%)	Controls Relapse Rate (N) (%)	Reference	Experimental Length of time
Outpatient Alcohol	15 (26)	15 (87)	Brown et al. [8]	Ten months
Outpatient Cocaine	15 (47)	15 (93)	Brown et al. [8]	Ten months
Inpatient Opioids Detox	29 (18)	NA	Blum et al. [9]	Four months
Outpatient Alcohol	61 (7)	NA	Chen et al. [10]	12 months
Outpatient Heroin	4 (0)	NA	Chen et al. [10]	12 months
Outpatient Alcohol	23 (22)	NA	Miller et al. [11]	12 months
Outpatient Alcoholics	21 (30)	NA	Miller et al. [11]	24 months
Outpatient Alcohol	600 (0)	NA	Blum et al. [12]	3 months**
Outpatient Bariatric	16 (18.2)	11 (82.2)	Blum et al. [13]	3 months
Outpatient Bariatric	130 (14,7)	117 (41.7)	Blum et al. [13]	24 months
Average RDS	91.4 (18.29)	158 (76)*	NA	11.4

**Table 3:** Comparison of KB220 variants and GLP-1 analogs

Pro-dopamine regulator (KB220 and variants)	GLP-1 analogs
The KB220 compound “normalized” abnormal electrophysiological measures of the brain reward network in both alcoholics and heroin dependency [21]. This suggested dopamine homeostasis.	GLP-1 analogs enhance the release of GABA in the brain reward circuitry and boosts dopamine transporter (DAT1) to significantly decrease dopamine in synapses and reduce dopamine release at the reward site. This is considered by some (erroneously) to induce dopamine homeostasis *but works only when dopamine is too high* [52].
A clinical trial with Synaptamine Complex Variant KB220^™^ via IV (with n = 600+ alcoholic patients) which resulted in significant reductions in RDS behaviors [11].	Evidence to reduce alcohol craving behavior in animals and a few human trials, not FDA approved [53].
Chronic symptoms were significantly lessened, as measured by the Chronic Abstinence Symptom Severity (CASS) Scale [12]. Paired sample t-tests of pre/post-treatment scales revealed a significant decrease (p = .00001) from treatment times: for somatic (t = 16.1), for emotion (t = 19.1), and for impaired cognition (t =14.9) [12]. At a two-year follow-up, 23 of the participants who underwent KB220 IV treatment (a minimum of five IV treatments over a seven-day period) and then the oral form of the compound for 30+ days: at six months, 91% (n = 21) remained sober and 82% (n = 19) were free of relapse; at one year, 82% (n =19) were sober, 78% (n = 18) had no relapse; at two years, 91% (n = 21) were sober and 70% (n = 16) had no relapse [12].	GLP-1 analogs and discovered that they may further improve cognitive performance via at least eight different molecular mechanisms [54]. There are no human studies to access relapse rates with Ozempic.
KB220, in comparison to the placebo, displays activation of the caudate brain area and possibly a leveling off of heroin-generated abnormal connectivity in the putamen (a region associated with emotionality [22].	Ex4 (basically Ozempic) into the nucleus of the solitary tract (NTS), which can inhibit alcohol’s acute effects (measured by locomotor stimulation) of nucleus accumbens dopamine release and subsequent consolidation of memory (as measured with the CPP paradigm) [55, 56].
KB220Z significantly augments, compared to placebo, functional connectivity in the reward system and higher areas within rats [23]. Functional connectivity, brain connectivity recruitment (perhaps due to neuroplasticity), and dopaminergic function throughout the brain reward network were found [23].	Exenatide (basically Ozempic) administration increased functional connectivity of the left (NTS, hypothalamus, and thalamus) in obese participants. This connectivity showed a positive correlation with hunger scores in all participants but greater so in those who were obese [57].
CJ is a 38-year-old who has a history of substance use disorder and comorbid attention deficit hyperactivity disorder (inattentive type), with chronic shopping and hoarding behaviors. After taking KB220 for four weeks, the patient reported a marked enhancement in mental status and various behaviors, including a decrease in shopping and hoarding. A lifelong history of nightmares was eradicated. Her locus of control shifted toward internal [24].	No studies have been published employing Ozempic on shopping and hoarding.
KB220Z (via SQ and IP) strikingly and instantaneously reduced binge drinking of a solution of 10 % ethanol in rats (females and males) [25]. There was no influence of SQ KB220Z on 3% sucrose ingestion. Elevated activity in the open field and duration in the open arm part of the EZM was reduced, reflecting reduced anxiety [25].	Semaglutide (Ozempic) dose-dependently reduced binge-like ETOH drinking in mice, as well as intake of noncaloric/caloric solutions. Semaglutide also reduced dependence-induced and binging of ETOH consumption in rats. Semaglutide enlarged sIPSC frequency in both CeA and ILC neurons from alcohol-naive rats. This implied enhanced GABA release (which works by reducing dopamine release) [58].
KB220 was used to detoxify opioid-dependent patients instead of providing opioids. Out of 17 subjects, merely three received Buprenorphine/Naloxone (Bup/nx) in concert with KB220Z. Only three participants had a relapse while on KB220 in the initial two weeks of the study. The rest of the participants (82%) were sustained on KB220Z. The participants were maintained without any supplementary Bup/nx for 120 days and, in one specific participant, for 214 days, thus decreasing opioid-seeking [9].	The two primary output paths from the nucleus accumbens are (1) GABAergic medium spiny neurons (MSNs; D1R-expressing) and (2) D2Rs (MSNs). Activation of D1R-expressing MSNs encourages reward-seeking, whereas activation of D2R-expressing MSNs decreases reinforcement by causing adverse effects. GLP-1 receptors are expressed on both D1R- along with D2R-expressing MSNs. It is conceivable that exendin-4 attenuates opioid taking by antagonizing opioid-induced activation of D1R and also decreases opioid-seeking via inhibition of D2R MSNs [59].
JN was a 77-year-old and had a memory decline. Treatment with KB220 was tested using the Animal Naming Test (ANT). This resulted in greater scores (p = 0.04762) on the ANT. The patient’s pretest scores increased from the 30^th^ percentile to the 76^th^ percentile with a single administration to the 98^th^ percentile at six months with the second administration [26].	The GLP1 agonist liraglutide (similar to Ozempic) delayed or blunted the progressive memory decline associated with hippocampal neuronal loss in mouse models of pathological aging with traits of neurobehavioral and neuropathological impairment often observed in those with early stage/sporadic AD [60].
A 51-year-old obese woman with comorbid PTSD and depression with a suicide attempt was studied using KB220. Also, she experienced frightening nightmares related to her childhood sexual/physical abuse from her family (one perpetrator was her father with alcohol use disorder). After KB220, her frequency and type of dreams changed dramatically to happiness. A second patient, a 39-year-old female with PTSD, also suffered from frightening nightmares. KB220Z to diminish withdrawal symptoms from methadone and subsequently described positive dreams [27].	There is some evidence that GLP1 agonists significantly reduce depression [61]. Still, there is also the first reported case of a suicide attempt associated with liraglutide (a GLP-1 agonist) in a Japanese woman. She was 33 years old with a diagnosis of type 2 diabetes and was taking liraglutide (72 mg SQ) [62]. There are no published studies in PubMed about GLP1 stimulation and induction of effects related to nightmares.
Willuhn et al. reported that substance use, and also other addictive behaviors increased as dopaminergic function was reduced. Blum’s group assessed the use of KB220Z^™^ on the reward circuitry. The study included ten people with a heroin use disorder who were undergoing protracted abstinence (on average, 16.9 months), thus attenuating opioid intake. The randomized placebo-controlled crossover study divulged an increase in BOLD fMRI activation within the caudate-accumbens-dopaminergic pathways (KB220 vs. placebo) following a one-hour acute administration. The active compound decreased resting-state activity in the putamen of participants who were abstinent with heroin use disorder. This increased functional connectivity was detected in a neural circuit that comprised the dorsal anterior cingulate, nucleus accumbens, medial frontal gyrus, occipital cortex, posterior cingulate, and cerebellum [28].	There is little evidence in animal studies that GLP1-agonists may help to attenuate opioid intake, and currently, there are no human trials [63].
This was the first report to reveal the contribution of the PFC in the qEEG response to a D2 agonist (Synaptose Complex KB220Z^™^), which was most apparent in those with the A1 allele of the D2 dopamine receptor. In polydrug abusers, KB220Z^™^ prompted regulation of brain electrical. The results suggest a phase change in the electrical activity from low amplitude/power to a more regulated activity (with an increase of 6.169 mV) throughout the PFC [29].	There are no publications about GLP1 -agonists in regard to qEEG.
We present a case series of four patients with a striking and persistent assuagement of nightmares in those diagnosed with PTSD/ADHD and/or opioid use disorder. The cessation of such nightmares was seen in 10–12 months post-treatment without recurrence. Following the seeding of the brain regions of the dorsal hippocampus, they found enhanced connectivity across numerous regions of interest (with the exception of the PFC), displaying neuroplasticity [30].	There is no reported evidence for GLP1-agonists affecting nightmares.
In a male diagnosed with full-blown RDS, including sexual addiction and repetitive paraphilia, within one week of KB220z, the repetitive paraphilia was abolished. There were also several other positive effects, such as enhanced focus. This remained after discontinuing the compound, which suggested a neuroplastic change [31].	There is no reported evidence for GLP1-agonists affecting repetitive paraphilia.
A 72-year-old male with a diagnosis of ADHD was examined using low-resolution electromagnetic tomography (LORETA) at baseline and one hour after administration of the KB220z compound. Tasks including eyes-closed, open, and working memory were employed. Z-scores averaged across these revealed increases in various frequency bands within the anterior, dorsal, and posterior cingulate areas, as well as the right DL-PFC, during the working memory task with the compound [32].	There is no reported evidence for GLP1-agonists affecting ADHD.
Blum’s group reported that the qEEGs of patients: one with alcohol use disorder and one with heroin use disorder with abnormalities (i.e., pervasive theta and pervasive alpha activity, respectively) during prolonged abstinence (reduced heroin intake) are considerably normalized by the administration of a single IV dose of Synaptamine Complex Variant KB220^™^ [33].	There is some animal evidence that glp1 agonists can reduce heroin intake and even drug reinstatement in rats [64].
The main ingredient in the patented KB220 is d-phenylalanine (DPA), an inhibitor of carboxypeptidase prolonging enkephalin activity. Blum’s group observed a significant attenuation of both forced and volitional ETOH intake, respectively, by both acute and chronic therapy with hydrocinnamic acid (a metabolite of DPA) and D-phenylalanine [34].	While some evidence shows that exenatide (Ozempic) evoked β-endorphin release from cultured microglia and in the spinal cord, no studies connect this effect on endorphins and enkephalins to antiaddiction [65].
L-DOPA amplifies ethanol-induced narcosis in mice [18]. The enhancement of monoamines by L-DOPA administration may be the reason. This work indicates the importance of dopamine and alcoholism [35]. ETOH augmentation	(1) Liraglutide (GLP-1 agonist) had an anti-anxiety effect; (2) augmented the anti-anxiety properties of ETOH; (3) thwarted development tolerance to ethanol’s anti-anxiety properties; and (4) thwarted anxiety associated with withdrawal [65]. ETOH augmentation
Blum’s group completed a study using SAAVE KB220 in a residential program for alcohol use disorder in a double-blind protocol for 30 days (n = 22). The compound increases monoamines, enkephalins, and GABA, all of which might be functionally deficient in alcohol use disorder. The compound, versus placebo, (1) had a decreased building up to drink score (1 vs. 2) (2) which did not require benzodiazepines PRN (0% vs. 94%), (3) discontinued tremors sooner (72h. vs. 96h.), and (4) did not lead to severe depression (0% vs. 24%) as measured on the MMPI [36].	There are no PubMed-listed studies linking GLP1-agonists and decreased benzodiazepine use on ETOH withdrawal.
Blum’s group completed a second study with the SAAVE KB220 compound in a residential program for those with alcohol use disorder and polydrug users in a double-blind protocol for 30 days (n = 62). Participants on the compound (vs. placebo) showed a decreased skin conductance level, which divulged a decreased stress response; they had enhanced Bess and Physical scores; and post detoxification, they had a sextuple decrease in leaving against medical advice (AMA) [37].	There are no PubMed-listed studies linking GLP1-agonists and AMA rates in residential treatment.
Blum’s group completed a third study with the SAAVE KB220 compound variants in a residential program for those with cocaine use disorder (Tropamine: compound variant stimulant abuse), alcohol use disorder (SAAVE: compound variant for alcohol abuse), and no compound for 30-days (n = 54). AMA rates for groups varied: Tropamine (4.2%), SAAVE (26.6%), and no compound (37.5%). Additionally, Tropamine reduced the craving for stimulants [38].	One acute, not chronic, study did not find evidence that low dose exenatide modified self-administration or the subjective effects of cocaine in those with CUD [67]. In a chronic study, administration of exendin-4 reduced cocaine-induced elevation of striatal dopamine c-fos expression, which implicated central GLP-1 receptors in these responses [68]. While the authors suggest this is a form of inducing DA homeostasis, this is incorrect genetically in cases of hypodopaminergic.
Blum’s group completed a fourth study with the SAAVE KB220 compound in an outpatient 10-week program for DUIs (SAAVE: AUD; Tropamine: CUD; n = 60). The compounds promoted recovery rates and reduced relapse rates (percentages given respectively): AUD (73%; 26%) and CUD (53%; 47%) compared to known rates of relapse (87–93%) [39].	There are no published studies involving the role of GLP1 agonists and Driving Under the influence (DUI).
In another study by Blum’s group, carbohydrate binge-eaters (n = 27) were given PCAL-103 (KB220 variant) in a 90-day outpatient program at a bariatric clinic. Sixteen patients received the compound (vs. n = 11 controls). The mean weight loss was 26.92 lbs. (vs. controls at 10 lbs.) Relapse rates were significantly less in the compound group (18.2%) versus the control group (81.8%) [40].	As of 11-16-23, there were 101 articles listed using the word search term “GLP1 agonists and weight-loss” [69].
This is the initial report in humans of the effects of daily ingestion of a particular amino acid mixture, Kantrol [KB220], on cognitive event-related potentials (ERPs) related performance. Cognitive ERPs were created by two computerized visual attention tasks: the Spatial Orientation Task (SOT), and Contingent Continuous Performance Task (CCPT), in typical young adult participants (acting as their own controls) before and after approximately 30 days of compound ingestion. A statistically significant amplitude augmentation of the P300 component of the ERPs [focus] was seen after KB220 for both tasks, as well as improvement with respect to cognitive processing speeds, indicating enhanced focus [41].	There are no studies linking GLp1-agonists and cognitive event-related potentials (ERPs), Spatial Orientation Task (SOT), and Contingent Continuous Performance Task (CCPT).
In a state psychiatric hospital, 12 CUD patients were enrolled in a randomized, double-blind, placebo-controlled study using a KB220 variant (similar to Tropamine: n =8) vs. control (placebo: n = 4). The results divulged cocaine craving decreased with the compound vs. control [42].	Studies classified a central mechanism where Ex-4 diminished cocaine seeking. This emphasizes GABAergic GLP-1R-expressing networks within the midbrain where anti-craving pathways [70]. This effect is akin to psychological extinction, only with a different mechanism than Naltrexone.
In a study, amino acid precursors paired with an enkephalinase inhibitor were used with six female patients with a history of eating disorders (three were comorbidly chemically dependent). All participants claimed initial benefit, with only one participant relapsing at six months. Additional data was collected with more patients with a history of eating disorders (n = 100), with 98% claiming improved mood and reduced cravings [43].	While it has been hypothesized that GLP1-agonists may have a positive effect on eating disorders, there is a paucity of research in the literature [71].
In a one-year outpatient program, Blum’s group conducted a study using Synaptamine ^™^ (a KB220 variant) in SUD/RDS. Participants (n = 76; 31 females, 45 males) with a mean age of 33 years had diagnoses of SUD. At one year of treatment, various measures showed significant mean decreases: anger (p < 0.001), depression (p < 0.001), stress (p = 0.002), anxiety (p < 0.001), and drug craving (p < 0.001), and scores of building up to relapse (p <0.001). With regard to recovery, energy (p < 0.001) and drug-seeking refraining (p < 0.001) both increased also at one year [44].	There are no studies linking GLP1-agonists to anger in humans, but in male mice, chronic treatment with Ex4 reduced aggression (dose-dependent), possibly by reducing dopaminergic activity [72].
Blum’s group wanted to test the combination of a narcotic antagonist with an enkephalinase inhibitor (DLPA) and other amino acid precursors to promote dopamine release in methadone-maintained patients (n = 12, compared to controls n = 1,000) to assist with compliance. The experimental group received a combination of Trexan and KB220 and was free of relapse for a mean of 262 days, which was significantly greater (p < 0.0001) than the controls [45].	Drugs of abuse can increase the frequency and magnitude of brief (1–3s), high-concentration (phasic) dopamine release events in terminal regions. Results suggest that GLP-1R activation exerts its effect by suppressing dopamine release [73].
Blum’s group conducted their first Precision Behavioral Management (PBM^®^) study using a DNA with a customized compound formulation (Geno-Trim, a KB220 variant) in 24 participants with obesity (due to RDS) completed a questionnaire [46].	The expression profile of eight T2D-related circulating miRNAs were assessed in people with diabetes (n = 26) with a GLP1-RA. Findings allude that miRNA expression might forecast the possibility of treatment response to various GLP1-RA agents [74].
Blum’s group examined chromium picolinate (CrP: an ingredient in the KB220 compound; n = 60) versus placebo (n = 62) for use in those with obesity (total n = 122) in those tested for variants of the DRD2 genes (A1 and A2). Carriers of the double A2 allele experienced significant weight loss. Carriers of a single or double copy of A1 showed no changes. The authors theorized that D2 receptor density interacted with CrP treatment and weight loss [47].	In cases of individuals with diabetes, obesity, and hyperglycemia, a glucagon-like peptide 1 (GLP-1) receptor agonist is recommended [75]. Moreover, numerous in vitro and in vivo experiments revealed that chromium supplements, particularly niacin-bound chromium (chromium-nicotinate), can be effective in assuaging insulin resistance as well as lowering plasma cholesterol and glucose levels [76].
Based on the scientific/medical literature, numerous candidate genes have been found that correlate with obesity, including D2 dopamine receptor, serotonin receptor (5-HT2a), methylenetetrahydrofolate reductase (MTHFR), Peroxisome Proliferator-Activated Receptor gamma (PPAR-y) and Leptin (OB) genes. One research study examined these five polymorphisms to develop a DNA-customized KB220 variant (LG839). Within this study, 21 participants were examined for differences in BMI at baseline and post-treatment (mean 41 days, up to 2,870 days). The weight loss for the participants was significant [48].	To the extent of our knowledge, there has been no attempt to develop a genetically customized GLP1 agonist.
Another study used LG389 in concert with genotypes (n = 1,058). A subset of this group (n = 27) of Dutch ancestry demonstrated significant weight loss and associated significantly lessening sugar cravings, snacks, late-night eating, and an increase in appetite suppression, sleep, happiness, and energy. Those with the A1 allele of the DRD2 correlated with an increase in days of continuing treatment [49].	Using PubMed and the search terms “GLP1 agonists and weight loss,” there are 1,037 articles as of 11-16-23.
Dopamine has been called the “anti-stress molecule.” In a study with Blum’s group, they examined the use of a KB220 variant (Synaptamine) for reducing anxiety in those with AUD and SUDs (polydrug users). This was a double-blind, placebo-controlled study. Those on the compound (n = 28) vs. placebo (n = 22) were measured as having significantly reduced stress based on SCL [50].	There were no double-blind studies showing GLP1’s anti-stress effects in PubMed using the terms “glp1 agonists and stress reduction in humans only” as of 11-16-23.
One study looked at heroin withdrawal and detoxification with the coadministration of various amino acids/precursors (5-HTP, L-Tyrosine, L-Glutamine, and Lecithin; n = 41) versus placebo (n = 42). For those in the treatment group (versus placebo), there was a significant decrease in tension-anxiety, depression-dejection, anger-hostility, disturbance in mood, fatigue-inertia, and an increase in vigor-activity [51].	There are no human studies on the incorporation of GLP1 agonists in SUD detoxification found in PubMed as of 11-16-23
